# Integrated genomics elucidates relative spatial homogeneity of embryonal brain tumors

**DOI:** 10.1186/2194-7791-2-S1-A10

**Published:** 2015-07-01

**Authors:** Marc Remke, Florence Cavalli, A Sorana Morrissy, Vijay Ramaswamy, David Jones, Roger Packer, Eric Bouffet, Gary Bader, Arndt Borkhardt, Stefan Pfister, Nada Jabado, Marco Marra, Michael D Taylor

**Affiliations:** Hospital for Sick Children, Toronto, Ontario Canada; University Hospital Düsseldorf (UKD) and German Cancer Consortium (DKTK), Düsseldorf, Germany; German Cancer Research Center (DKFZ), Heidelberg, Germany; Children's National Medical Center, Washington, D.C USA; University of Toronto, Toronto, Ontario Canada; the McGill University Health Center Research Institute, McGill University and, Montreal, Quebec Canada; BC Cancer Agency, Michael Smith Genome Sciences Centre, Vancouver, British Columbia Canada

## Introduction

Genome-wide profiling and next-generation based sequencing studies have dramatically improved our understanding of embryonal brain tumor (EBT) biology in the recent years. However, the vast majority of these studies are based on the assumption that single biopsies are representative for the entire primary tumor. Intratumor heterogeneity constitutes a common phenomenon previously described in renal cell carcinoma (RCC) and high-grade glioma (HGG). Highly disparate molecular profiles of spatially separated tumor areas within the same tumor may preclude development of molecularly targeted therapies based on single tumor biopsies.

## Material and methods

To address this issue, we conducted multiregion whole exome sequencing, high-resolution DNA copy number analysis (Cytoscan HD), and transcriptional profiling on 39 distinct pediatric and adult tumors with a median of six spatially distant biopsies per tumor (range 4-11). Histological entities included AT/RT (n = 2), HGG (n = 17), medulloblastoma (n = 9), medulloepithelioma (n = 1), and RCC (n = 10). We assessed the degree of intratumor heterogeneity and subgroup affiliation using integrated genomics approaches.

## Results

Embryonal brain tumors demonstrated highly spatially homogenous transcriptomes. In contrast to adult glioblastoma, we showed that subgroup affiliation was stable in multiregion biopsies from the same medulloblastoma patient. Furthermore, EBT displayed highly similar focal and broad DNA copy number alterations compared to HGG and RCC. Multiregion sequencing further reinforced the relatively higher degree of intratumor homogeneity in EBT. Compared to HGG or RCC, somatic mutations in EBT were much more likely to be ubiquitous throughout the tumor.

## Conclusions

The relative spatial homogeneity of EBT suggests that limited biopsies are representative of the tumor genomics landscape, which has important implications for biological classification and development of targeted therapies for these tumors.

